# Structure-Aware Multi-Animal Pose Estimation for Space Model Organism Behavior Analysis

**DOI:** 10.3390/ani15213139

**Published:** 2025-10-29

**Authors:** Kang Liu, Shengyang Li, Yixuan Lv, Rong Yang, Xuzhi Li

**Affiliations:** 1 Technology and Engineering Center for Space Utilization, Chinese Academy of Sciences, Beijing 100094, China; liukang@csu.ac.cn (K.L.); lvyixuan@csu.ac.cn (Y.L.); yangrong23@csu.ac.cn (R.Y.); xzhli@csu.ac.cn (X.L.); 2 Key Laboratory of Space Utilization, Chinese Academy of Sciences, Beijing 100094, China; 3 University of Chinese Academy of Sciences, Beijing 100049, China

**Keywords:** multi-animal pose estimation, microgravity, space biology, *C. elegans*, zebrafish, *Drosophila*, keypoint detection, behavioral analysis

## Abstract

Multi-animal pose estimation is a critical technique for enabling fine-grained quantification of group animal behaviors, which holds significant scientific value for uncovering behavioral changes under space environmental factors such as microgravity and radiation. Currently, the China Space Station has conducted a series of space biology experiments involving typical model organisms, including *Caenorhabditis elegans* (*C. elegans*), zebrafish, and *Drosophila*. However, substantial differences in species types, body scales, and posture dynamics among these animals pose serious challenges to the generalization and robustness of traditional pose estimation methods. To address this, we propose a novel, flexible, and general single-stage multi-animal pose estimation method. The method constructs species-specific pose group representations based on anatomical priors, incorporates a multi-scale feature-sampling module to integrate shallow and deep visual cues, and employs a structure-guided learning mechanism to enhance keypoint localization robustness under occlusion and overlap. We evaluate our method on the SpaceAnimal dataset—the first public benchmark for pose estimation and tracking of model organisms in space—containing multi-species samples from both spaceflight and ground-based experiments. Our method achieves AP scores of 72.8%, 62.1%, and 67.1% on *C. elegans*, zebrafish, and *Drosophila*, respectively, surpassing the state-of-the-art performance. These findings demonstrate the effectiveness and robustness of the proposed method across species and imaging conditions, offering strong technical support for on-orbit behavior modeling and large-scale quantitative analysis.

## Simple Summary

In space biology experiments, accurately estimating the body poses of small animals—such as *Caenorhabditis elegans*, zebrafish, and *Drosophila*—under complex conditions is essential for understanding how microgravity and other space-related factors affect behavior. This study proposes a deep learning-based multi-animal pose estimation method tailored to challenging scenarios involving dense populations, diverse postures, and frequent occlusions. By integrating anatomical priors, multi-scale visual features, and a structure-guided learning mechanism, the method demonstrates robust keypoint localization even under overlapping or partially occluded conditions. It supports end-to-end inference without the need for separate object detection or instance grouping steps, and achieves higher efficiency than traditional top–down and bottom–up approaches. The method was evaluated on the public SpaceAnimal dataset, which includes three types of space-experiment animals. Results show that it consistently outperforms existing baselines in pose estimation accuracy while maintaining a good balance between accuracy and efficiency. These findings highlight the method’s potential to enable automated, fine-grained behavior analysis in space life science research. As this study used only publicly available video data from previous space missions, no new animal experiments were conducted, and no ethical approval was required.

## 1. Introduction

Space life science is a vital branch of space research that aims to investigate how organisms behave, develop, and adapt under extreme environmental conditions, such as microgravity and radiation [1,2]. With the advancement of space experimental platforms and transportation capabilities, an increasing number of model animals have been sent into orbit [3]. Studying their behavioral changes in space helps reveal mechanisms of neural regulation, motor control, and environmental adaptation [4]. Compared to traditional metrics, such as gross locomotor activity or trajectory analysis, skeleton-based fine-grained behavior analysis offers high temporal and spatial resolution, supporting hierarchical modeling from individual motion to group interactions, and from posture transitions to behavior categories [5,6]. It plays a key role in building in-orbit behavioral atlases and quantifying environmental effects. Therefore, developing a robust and generalizable animal-pose-modeling framework is essential for advancing space life science from observational studies to mechanistic analysis [7].

Multi-animal pose estimation aims to detect the 2D skeletal keypoints of all animals present in a single image and serves as the critical upstream task for fine-grained behavior modeling [5,8,9]. Its primary goal is to accurately and reliably localize anatomical landmarks of each individual, providing a structured foundation for downstream tasks such as pose tracking, identity recognition, action understanding, and behavior classification [10,11]. In high-density, diverse animal populations often encountered in space experiments, the quality of pose estimation directly determines the interpretability and reliability of behavior analysis. For example, head–tail oscillation frequencies of *C. elegans* can be used to infer motor function degradation; zebrafish posture sequences can help identify disoriented swimming behaviors under microgravity; and *Drosophila* skeleton sequences can support the analysis of motion deficiency as well as high-level behaviors such as grooming, crawling, flying, courtship, and mating in space environments.

In recent years, deep learning has driven remarkable progress in animal pose estimation [12,13,14,15,16]. Representative tools such as DeepLabCut [5], LEAP [6], and SLEAP [8] have been widely adopted in ground-based studies involving rodents and small invertebrates. These tools primarily adopt two-stage strategies—first detecting individuals, then regressing keypoints—or bottom-up pipelines involving keypoint grouping. However, they encounter significant challenges in scenarios involving large animal populations, scale variations, highly flexible postures, and severe occlusions. In space biology experiments, to reduce the risk of experimental failure due to in-orbit mortality, it is common to deploy a greater number of individuals per imaging field, exceeding the design assumptions of existing frameworks. Additionally, animals in microgravity may exhibit unconventional motion patterns [17,18], further complicating modeling and generalization.

To address these challenges, we propose a flexible and general single-stage multi-animal pose estimation framework, capable of modeling animals with diverse species, scales, and postural variations. Specifically, we design a two-hop regression architecture, which first predicts intermediate part points before regressing final keypoint locations, allowing the model to infer spatial relations through both direct and indirect connections. This design bypasses instance detection and grouping, thereby improving efficiency and enabling end-to-end learnability. In addition, we construct a biologically informed pose-grouping representation, which organizes keypoints according to anatomical regions (e.g., head, back, wings, and abdomen). This biologically guided partition enhances feature interpretability and facilitates adaptation across different species. Furthermore, we introduce two key components: a Multi-scale Feature Sampling Module to extract fine-grained visual cues at keypoint locations across varying body sizes, and a Structure-guided Learning Module to model flexible inter-keypoint dependencies and ensure robustness under occlusion and crowding.

The main contributions of this work are summarized as follows:

- We propose a single-stage two-hop regression framework for multi-animal pose estimation that avoids dependence on object detection and post-processing, achieving both high efficiency and precision.
- We design anatomical prior-based pose grouping representations tailored to *C. elegans*, zebrafish, and *Drosophila*, enabling structurally aware modeling across species.
- We develop a Multi-scale Feature Sampling Module to enhance spatial feature representation and adaptability across animals of different body scales.
- We introduce a Structure-guided Learning Module that captures inter-keypoint structural relationships, significantly improving robustness under occlusion and overlap conditions.
- We validate our method on a *C. elegans* dataset collected from actual missions aboard the China Space Station, and conduct extended experiments on zebrafish and *Drosophila* datasets to demonstrate generalizability and state-of-the-art performance.

## 2. Related Work

Pose estimation serves as a fundamental step in fine-grained animal behavior analysis, enabling the extraction of spatial features that support accurate behavior modeling and interpretation. With the advancement of deep learning, the analysis of animal behavior has transitioned from traditional manual observation to automated, data-driven approaches. Deep learning-based pose estimation frameworks have become increasingly influential across various domains, including neuroscience, ethology, and developmental biology [19,20]. Furthermore, these methods have demonstrated practical value in real-time applications, such as scientific research, precision agriculture, and wildlife monitoring [21,22,23].

Most pose estimation methods can be broadly categorized into the following groups: top–down, bottom–up, and single-stage regression.

### 2.1. Top–Down Methods

Top–down methods typically begin by detecting individual objects and then perform keypoint localization within each bounding box [24,25,26,27,28]. Representative approaches include CPM [24], which applies intermediate supervision for stepwise refinement; Hourglass [25], which utilizes a symmetric encoder–decoder structure for multi-scale feature fusion; and HRNet [26], which maintains high-resolution representations throughout the network. To enhance robustness against inaccurate bounding boxes, RMPE [29] introduces spatial transformer modules and pose-guided proposal generation. Building on these foundations, DCPose [27] and pose-warping methods [28] further incorporate temporal consistency to mitigate errors caused by inter-frame occlusion or missing keypoints. Although some of these methods improve robustness in mildly occluded scenarios by leveraging spatial or temporal cues, top–down approaches are inherently inefficient in dense scenes due to the need for repeated detection and cropping per instance. Moreover, their strong reliance on detector performance makes them prone to error accumulation, and the computational cost increases significantly with the number of individuals—limiting their applicability in high-density, multi-animal scenarios such as those encountered in space experiments.

### 2.2. Bottom–up Methods

Bottom–up methods detect all keypoints in an image without relying on instance-level detection, and then group them into individual poses, offering higher efficiency and better scalability in crowded scenes. These methods are typically categorized into keypoint grouping and center-based regression approaches [30,31,32,33,34,35,36]. Keypoint-grouping methods, such as DeepCut [37], OpenPose [30], and Associative Embedding [31], leverage pairwise affinities or embedding similarities to associate detected keypoints. Jin et al. [32] further proposed Differentiable Graph Grouping to enable end-to-end trainable keypoint association. In contrast, center-based methods like PifPaf [33] and Point-Set Anchors [34] regress offset vectors from keypoints to their corresponding instance centers for pose grouping. Luo et al. [35] revisited the heatmap regression paradigm and introduced scale- and weight-adaptive strategies to improve robustness against scale variance and labeling ambiguity, enabling bottom–up methods to match top–down accuracy while preserving efficiency. Despite these advances, bottom–up pipelines still struggle with fine-grained localization and reliable keypoint grouping under occlusion or dense overlaps.

### 2.3. Single-Stage Regression Methods

Recently, single-stage regression methods that directly predict keypoint coordinates have gained traction due to their improved computational efficiency and simpler pipeline. These methods bypass both object detection and grouping stages, offering a more streamlined alternative. Representative approaches include DEKR [36], AdaptivePose [38], and CID (Contextual Instance Decoupling) [39]. However, most of these models were designed and benchmarked on human pose estimation datasets, and their generalization to animal datasets—particularly those involving dense populations and severe occlusion—remains largely unexplored. Empirical evidence suggests that their performance in such challenging scenarios is limited.

### 2.4. Animal-Specific Pose Estimation Studies

To meet the growing needs of behavioral analysis in model organisms, many frameworks have extended human pose estimation techniques to animals. DeepLabCut [5], LEAP [6], and DeepPoseKit [13] enable efficient single-animal pose estimation with minimal supervision. SLEAP [8] further supports multi-animal settings by combining top–down and bottom–up pipelines with identity tracking. Other tools, such as OpenMonkeyStudio [12], MARS [14], and multi-animal DeepLabCut [15], address 3D estimation, behavior recognition, and group tracking. Recent studies also integrate pose with neural signals to analyze social cognition in naturalistic contexts [16].

Several benchmark datasets support this progress. Animal Kingdom [40] provides large-scale pose annotations across species and environments. MultiFly [41] focuses on *Drosophila* behavior with 21-keypoint labels under optogenetic control. MABe [42] emphasizes naturalistic multi-agent behavior and multi-task prediction. However, these datasets are collected under terrestrial conditions and do not reflect the challenges of space experiments, where limited onboard volume often increases animal density and occlusion. Microgravity further induces greater pose diversity and unpredictability, complicating keypoint localization and identity consistency. To address these issues, we previously released the SpaceAnimal dataset [43], which provides fine-grained pose annotations of *C. elegans*, Zebrafish, and *Drosophila* on the China Space Station. It presents multiple challenges, including dense scenes, diverse postures, and cross-species structural differences in scale.

These limitations highlight the difficulty of directly transferring ground-based methods to space scenarios. Although some studies have attempted to improve robustness through temporal modeling (e.g., DCPose) or keypoint association using graph convolutions (e.g., Differentiable Graph Grouping by Jin et al. [32]), existing approaches still struggle to ensure accurate keypoint localization and identity consistency in the presence of dense occlusion, frequent interactions, and structural variations among species in space experiments. To overcome these challenges, this paper introduces structure-aware modeling strategies to enhance the utilization of structural information, thereby improving the robustness and generalization of multi-animal pose estimation in space environments.

## 3. Methods

In this section, we first introduce the overall architecture of the proposed single-stage multi-animal pose estimation framework. We then describe the three core components in detail: the Pose-Grouping Representation, the Multi-Scale Feature Sampling (MFS) Module, and the Structure-Guided Learning (SGL) Module. Finally, we present the training objectives and corresponding loss functions.

### 3.1. Overall Architecture

We propose a single-stage framework tailored for multi-type space animal pose estimation. In contrast to traditional two-stage approaches—which rely on separate object detection (top–down) or complex keypoint grouping (bottom–up)—our method directly regresses keypoint locations in a fully differentiable, end-to-end manner.

To alleviate the challenge of directly regressing long-range displacements from instance centers to distant keypoints, we introduce a semantically guided two-hop regression strategy, inspired by AdaptivePose [38]. Instead of predicting the center-to-keypoint offset in a single step, we decompose it into two successive hops: (1) from the center point to an adaptive part point, and (2) from the part point to the corresponding keypoint. This decomposition enhances regression stability and supports more fine-grained feature learning, particularly for animals with highly deformable and variable body structures.

**Figure 1:**
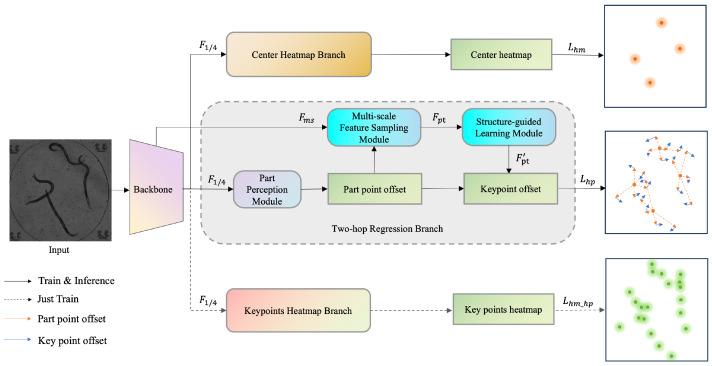
Overview of the proposed method. The backbone network extracts multi-scale features, which are then processed by three branches. The Two-Hop Regression Branch first applies the Part Perception Module to predict offsets from the center point to part points using features at $F_{1/4}$. Subsequently, the multi-scale features $F_{ms}$ are fed into the MFS and SGL modules to refine the part features $F_{pt}^{\prime }$, which are used to predict the second-hop offsets from part points to keypoints.

As illustrated in Figure 1, the input image is first processed by the HRNet backbone [26] to extract multi-scale semantic features $F_{ms}$, without relying on any pre-generated bounding boxes or instance-wise cropping. Unlike conventional top–down pipelines, our framework performs single-pass feature extraction and employs a shared global feature map for joint reasoning of instance and keypoint locations. The extracted features are then simultaneously processed by three specialized branches:

- Center Heatmap Branch: This predicts the heatmap of instance centers;
- Two-Hop Regression Branch: This predicts the offset vectors from centers to keypoints through intermediate part points;
- Keypoint Heatmap Branch: This assists geometric prior learning of keypoint distributions (used only during training).

In the Two-Hop Regression Branch, the predicted center locations serve as anchors to initiate the Part Perception Module, which regresses the first-hop offsets ${\vec{\mathrm{off}}}_{1}$ from each center to a set of adaptive part points $P_{parts}$. These part points are not predefined but are dynamically learned for each instance, enabling the network to capture semantically meaningful regions (e.g., head, body, and tail) according to the pose-grouping scheme described in Section 3.2.

Next, the proposed Multi-Scale Feature Sampling (MFS) Module extracts enriched features $F_{pt}$ at each part point position from the shared $F_{ms}$. These features are then refined by the Structure-Guided Learning (SGL) Module to produce structure-aware representations $F_{pt}^{\prime }$. Finally, the model regresses the second-hop offsets ${\vec{\mathrm{off}}}_{2}$ from part points to their corresponding keypoints $K_{keypoints}$.

The final keypoint location is obtained by summing the two learned offset vectors:

(1) $$\vec{\mathrm{off}}={\vec{\mathrm{off}}}_{1}+{\vec{\mathrm{off}}}_{2}$$

where ${\vec{\mathrm{off}}}_{1}$ represents the offset from the instance center $C_{center}$ to the part point $P_{parts}$, and ${\vec{\mathrm{off}}}_{2}$ denotes the offset from the part point to the corresponding keypoint $K_{keypoints}$.

Although the offset regression is conceptually decomposed into two stages, both components are jointly supervised using a single L1 loss with respect to the full center-to-keypoint displacement (see Equation (4)). This unified optimization enables end-to-end training and mitigates cumulative errors, unlike traditional cascaded regression schemes. Consequently, the part-aware regression design provides a more robust, interpretable, and biologically consistent alternative to direct regression.

The Keypoint Heatmap Branch is utilized only during training to enhance geometric representation learning and guide the backbone in encoding spatial priors more effectively. This auxiliary branch is removed during inference to maintain computational efficiency.

### 3.2. Pose-Grouping Representation Method

Distinct from previous approaches that represent animal poses solely by a collection of keypoints, we propose a novel pose-grouping representation that explicitly models hierarchical relationships among anatomical structures. This representation incorporates both keypoints and their corresponding parts, thereby capturing the internal structural organization of different species more effectively. In particular, we introduce part points to describe the intermediate structures between the instance center and keypoints. These adaptive part points serve to decompose long-range offsets from the center point to keypoints into shorter, semantically meaningful links, thus mitigating cumulative regression errors.

**Figure 2:**
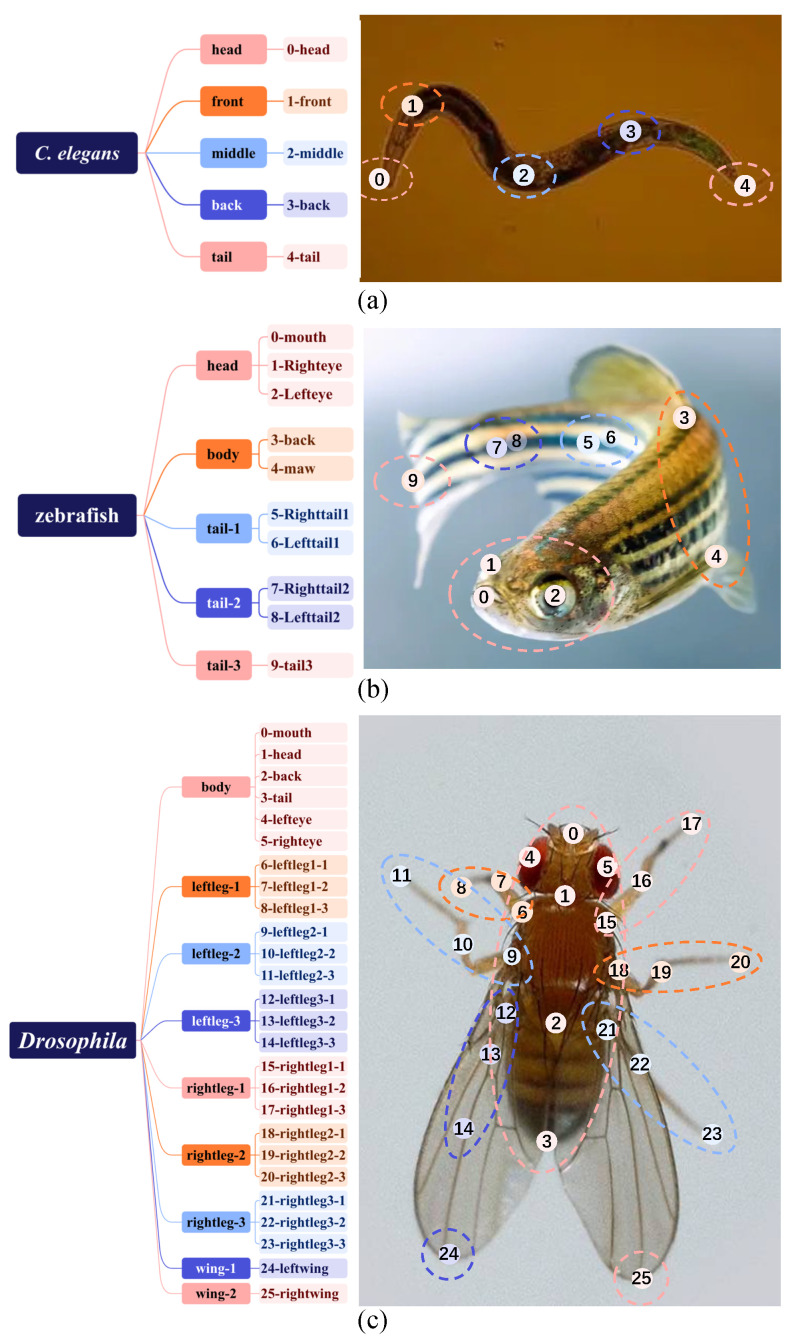
Visualization of the proposed pose grouping representation. Subfigures (**a**–**c**) correspond to *C. elegans*, zebrafish, and *Drosophila*, respectively. The tree diagram on the left illustrates the hierarchical organization of parts and their corresponding keypoints, while the right panels show examples of grouping representations, where dots denote keypoints and dashed lines indicate part connections.

Considering the anatomical characteristics and flexible postures of different organisms, we define species-specific grouping schemes: *C. elegans* is divided into five parts, each containing one keypoint (Figure 2a); zebrafish is divided into five parts, each containing one to three keypoints (Figure 2b); and *Drosophila* is divided into nine parts, each containing one to six keypoints (Figure 2c). In the tree diagrams of Figure 2, the root node corresponds to the animal instance, the first-level nodes correspond to body parts $P_{parts}$, and the second-level nodes represent the keypoints $K_{keypoints}$ associated with each part. Each part is represented by a part point that is dynamically regressed from the instance center $C_{center}$ using the Part Perception Module (see Figure 1). While the structural relationships among different parts are flexible and variable, the keypoint relationships within each part remain relatively stable. This hierarchical grouping representation guides the network to learn flexible yet interpretable pose structures while reducing model complexity.

The proposed representation establishes a three-level hierarchy from the instance center to parts and finally to keypoints, which naturally aligns with the two-hop regression mechanism introduced in Section 3.1. This hierarchical process can be formalized as follows:

(2) $$C_{center}\to P_{parts}\to K_{keypoints}$$

where $C_{center}$ denotes the instance center, $P_{parts}$ represents the set of part points obtained via regression from $C_{center}$, and $K_{keypoints}$ denotes the keypoints corresponding to each part.

### 3.3. Multi-Scale Feature-Sampling Module

Space model organisms, such as *C. elegans*, zebrafish, and *Drosophila*, exhibit considerable morphological diversity across genera. Observational images from in-orbit and ground-based experiments demonstrate significant variation in the scale of experimental subjects and the field of view, leading to different demands for feature extraction at multiple spatial resolutions. To robustly estimate poses for various space model organisms with diverse sizes and appearances, it is essential to extract discriminative features at appropriate spatial resolutions. Single-scale representations often fail to simultaneously capture fine-grained details and global context—especially in heterogeneous settings such as microgravity experiments, where object scales can vary drastically across species and imaging conditions. Motivated by this, we propose a Multi-Scale Feature Sampling (MFS) strategy that enables adaptive selection and fusion of hierarchical features, allowing the network to dynamically balance between texture-rich shallow layers and semantic-rich deep layers. This design enhances the network’s ability to handle both small, deformable organisms (e.g., *Drosophila*) and larger species (e.g., zebrafish) under varying field-of-view settings.

Traditional pose estimation models, such as DETR, CID, and AdaptivePose, typically retain higher-resolution features using a 1/4 downsampling stride in the backbone. While this design helps preserve spatial details, it limits adaptability to varying object scales. To address this limitation, we propose the Multi-Scale Feature Sampling (MFS) Module, which enables hierarchical sampling and adaptive fusion of multi-scale features tailored to the diverse appearance characteristics of different space animals.

**Figure 3:**
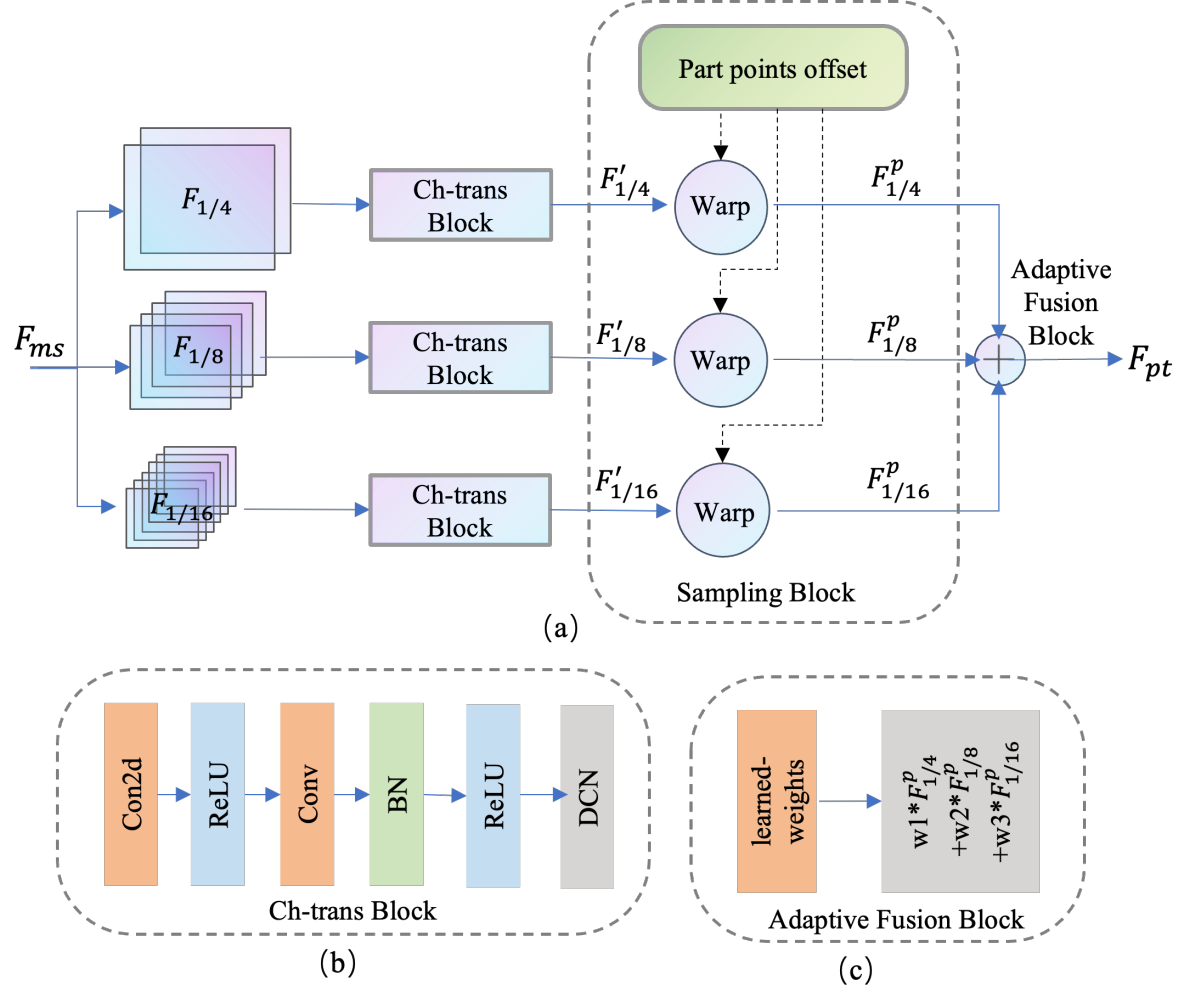
(**a**) The details of Multi-scale Feature Sampling Module (MFS). The input multi-scale features $F_{ms}$ are processed by HRNet backbone network, which includes $F_{1/4},\;F_{1/8},\;F_{1/16}$, et al. The multi-scale features are fed to the (**b**) Ch-trans Block separately, and then the part points offset is used to perform warp operation to sample the multi-scale features. The output $F_{pt}$ is obtained by weighting the sampled multi-scale features using the (**c**) Adaptive Fusion Block.

As shown in Figure 3, the HRNet backbone generates multi-scale features $F_{1/4},\;F_{1/8},\;$$F_{1/16},\ldots$ with varying spatial resolutions, capturing both fine and coarse information. To enable efficient fusion across different scales, each feature is first processed through a Channel Transformation Block (Ch-trans Block), which standardizes the channel dimensions. Furthermore, as illustrated in Figure 3b, we employ deformable convolutions (DCNs) to enhance spatial adaptability at each feature level. In addition, the subsequent multi-scale sampling and fusion operations are implemented using PyTorch 1.10’s efficient warp function, enabling fast differentiable sampling across scales. This design is particularly beneficial for modeling non-rigid and irregular morphologies of biological organisms while maintaining computational efficiency.

Next, we obtain transformed features $F_{1/4}^{\prime },\;F_{1/8}^{\prime },\;F_{1/16}^{\prime }$, which are sampled using predicted part point offsets. A bilinear interpolation (warp) operation is performed at the corresponding part locations to extract feature vectors. For example, for an instance $C_{\mathrm{inst}1}$ in the *C. elegans* dataset, the predicted part point set $\left\{P_{\mathrm{head}1},\;P_{\mathrm{front}1},\;P_{\mathrm{middle}1},\;P_{\mathrm{back}1},\;P_{\mathrm{tail}1}\right\}$ is used to sample features from each scale, yielding $\left\{F_{1/4}^{p},\;F_{1/8}^{p},\;F_{1/16}^{p}\right\}$.

These sampled features are fused through a learnable weighted summation using the Adaptive Fusion Block (see Figure 3c), enabling the model to adaptively emphasize the most informative scale for each part. The fused feature of each part point is denoted as $F_{pt}$, where each $F_{pt}$ corresponds to a predicted part point $P_{parts}$ obtained from the first-hop regression. Notably, this fusion is performed at the pixel level for all instances in parallel.

Multi-scale features bring complementary benefits: shallow features with higher resolution contain richer texture details and are beneficial for localizing fine structures in small animals; deeper features with larger receptive fields carry richer semantics, which are essential for handling occlusions and pose estimation in complex or large-scale subjects. By leveraging features across scales and depths, MFS significantly enhances the robustness and generalizability of pose recognition across species and experimental scenarios.

### 3.4. Structure-Guided Learning Module

To effectively estimate the poses of flexible and deformable organisms in space experiments—such as *C. elegans*, zebrafish, and *Drosophila*—it is crucial to model the structural dependencies among anatomical parts, especially under frequent occlusion and overlapping conditions. These organisms exhibit non-rigid motion patterns and diverse topologies: *C. elegans* lacks a rigid skeleton and often coils or overlaps; zebrafish display highly dynamic tail flicks; and *Drosophila*’s limbs and wings frequently occlude each other. Such characteristics challenge conventional regression-based methods, which typically treat keypoints independently. However, existing approaches, such as DEKT, CID, and AdaptivePose, do not explicitly capture structural correlations between parts. This often leads to failures in resolving ambiguous or overlapping poses, particularly in crowded views or low-quality image frames.

To address these challenges, we introduce the Structure-Guided Learning (SGL) module. It leverages a Transformer-based self-attention mechanism to learn the internal structure of each instance by encoding inter-part relationships. This enhances the network’s structural perception and enables it to acquire animal-specific pose priors, thereby improving robustness and accuracy under complex spatial conditions.

**Figure 4:**
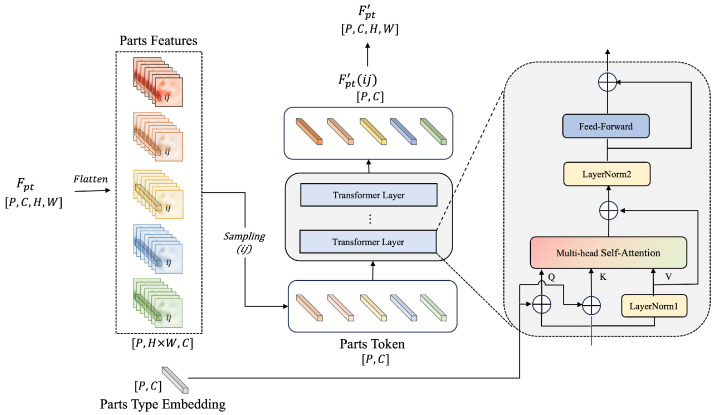
Details of the Structure-Guided Learning Module (SGL). Part features $F_{pt}$ are first flattened and sampled at pixel points (i,j). A part-type embedding is combined with the part token sequence and passed into stacked Transformer layers to compute multi-head self-attention. The query and key are derived from the sum of part tokens and type embeddings, while the value is obtained through a linear transformation of the part tokens. The final enhanced features $F_{pt}^{\prime }$ encode structural relationships among parts.

As shown in Figure 4, the part feature tensor $F_{pt}$ with shape [P,C,H,W] is first flattened into [P,H×W,C], where *P* is the number of parts, *C* is the feature dimension, and *H* and *W* denote the spatial resolution of the feature map. For each spatial location (i,j), we sample part features to construct an instance-level representation of shape [P,C].

Meanwhile, a learnable part-type embedding matrix of shape [P,C] is initialized. The sampled part features (referred to as part tokens) represent each anatomical region of an instance—for example, for *C. elegans*, the set $\left\{P_{\mathrm{head}},P_{\mathrm{front}},P_{\mathrm{middle}},P_{\mathrm{back}},P_{\mathrm{tail}}\right\}$ corresponds to five body segments.

The sequence of *P* part tokens is then input into a Transformer architecture composed of multiple stacked layers. Each layer contains LayerNorm, Multi-Head Self-Attention (MHSA), LayerNorm, and a Feed-Forward Network (FFN). The query *Q* and key *K* are computed by summing the part tokens with the part-type embeddings, while the value *V* is derived from the part tokens through a learnable linear projection.

The multi-head attention is defined as follows:

(3) $$\mathrm{Attention}\left(Q,K,V\right)=\mathrm{softmax}\left(\frac{QK^{T}}{\sqrt{C}}\right)V$$

Through this mechanism, the network captures the structural relationships among body parts within each instance. The resulting output $F_{pt}^{\prime }\left(i,j\right)$ represents structurally enhanced features at pixel location (i,j). During inference, this computation is performed in parallel across all spatial positions of the feature map, producing a refined feature representation $F_{pt}^{\prime }$ that encodes both spatial and structural priors.

### 3.5. Loss Function

According to Figure 1, the overall framework comprises three branches: the Center Point Heatmap Branch, the Two-hop Regression Branch, and the Keypoint Heatmap Branch.

Center Point Heatmap Loss. The Center Point Heatmap Branch predicts the center position of each instance. The loss is calculated using the focal loss with penalty terms, as shown in Equation (4):

(4) $$L_{\mathrm{hm}}=\frac{1}{N}\sum_{n=1}^{N}\left\{\begin{array}{cc} {\left(1-{\bar{P}}_{c}\right)}^{\alpha }\log\left({\bar{P}}_{c}\right), & \mathrm{if}\;P_{c}=1\\ {\left(1-P_{c}\right)}^{\beta }{\bar{P}}_{c}^{\alpha }\log\left(1-{\bar{P}}_{c}\right), & \mathrm{otherwise} \end{array}\right.$$

where *N* is the number of positive samples, and ${\bar{P}}_{c}$ and $P_{c}$ are the predicted and ground truth confidence scores, respectively. Following [38], the hyperparameters are set as α=2 and β=4.

Two-hop Regression Loss. The Two-hop Regression Branch predicts the keypoint offset from the center. The regression loss is calculated using L1 Loss as in Equation (5):

(5) $$L_{\mathrm{hp}}=\sum_{n=1}^{K}\left|\bar{{\mathrm{off}}^{n}}-{\mathrm{off}}_{\mathrm{gt}}^{n}\right|$$

where ${\mathrm{off}}_{\mathrm{gt}}^{n}$ is the ground truth offset from the center to the *n*-th keypoint.

Keypoint Heatmap Loss. The Keypoint Heatmap Branch evaluates the pixel-level keypoint location. The loss is computed using a focal loss similar to the center heatmap, as in Equation (6):

(6) Lhm_ hp=1N∑k=1K(1−P¯c)αlog(P¯c),ifPc=1(1−Pc)βP¯cαlog(1−P¯c),otherwise

where *K* is the number of positive keypoint samples. Other parameters are the same as those used in Equation (4).

Total Loss. The final loss is computed as the weighted sum of the above three losses:

(7) Ltotal=λhmLhm+λhpLhp+λhm_ hpLhm_ hp

where $\lambda_{\mathrm{hm}}$, $\lambda_{\mathrm{hp}}$, and λhm_ hp are all set to 1, following the same configuration as the baseline AdaptivePose [38].

## 4. Materials

### 4.1. Datesets

All experiments in this study are conducted on the publicly available *SpaceAnimal* dataset [43], which was collected from biological experiments aboard the China Space Station. This dataset is specifically curated for multi-animal pose estimation and tracking under microgravity conditions, characterized by high-density populations, complex inter-individual interactions, and notable morphological diversity across species.

It comprises three representative model organisms—*C. elegans*, zebrafish, and *Drosophila*—with species-specific keypoint annotations that reflect their distinct anatomical and behavioral features. In particular, five keypoints are defined for *C. elegans* to capture the full-body curvature from head to tail; ten keypoints for zebrafish, covering the head, dorsal, ventral, and caudal regions; and twenty-six keypoints for *Drosophila*, including the head, thorax, abdomen, wings, and six legs.

Compared to conventional ground-based datasets, SpaceAnimal presents greater challenges such as dense spatial distributions, frequent occlusions, inter-body overlap, and significant inter-species scale variations, all of which exacerbate the quantization errors in heatmap-based localization. These attributes make it a highly demanding and valuable benchmark for evaluating structural modeling accuracy and cross-species generalization in space-oriented multi-animal pose estimation.

In all experiments, we follow the official train/test splits provided by the SpaceAnimal benchmark to ensure reproducibility and fair comparisons.

### 4.2. Implementation Details and Metrics

All experiments are implemented using PyTorch [44] and conducted on two NVIDIA RTX 3090 GPUs. HRNet-W32 [26], pre-trained on ImageNet [45], serves as the backbone across all experiments.

Training: We adopt the Adam optimizer with a weight decay of $1\times 10^{-4}$. Data augmentation strategies include random rotation ($\left[-30^{\circ },30^{\circ }\right]$), scaling ([0.75,1.5]), translation ([−40,40]), and horizontal flipping with a probability of 0.5. Image cropping and augmentation are applied to form training samples.

The input resolution is set to 512×512 for the *C. elegans* and zebrafish datasets, and 640×640 for the *Drosophila* dataset. The batch size is set to 16 for the *C. elegans* and zebrafish datasets, and 10 for the *Drosophila* dataset. For *C. elegans* and zebrafish, the learning rate is initialized as $1.25\times 10^{-4}$ and decayed by a factor of 10 at the 90th and 120th epochs, with training terminating at the 140th epoch. For *Drosophila*, training is conducted for 280 epochs, with learning-rate drops at the 230th and 260th epochs.

The learning-rate schedule follows a cosine annealing strategy, defined as follows:

(8) $$\eta_{t}=\eta_{\min}+\frac{1}{2}\left(\eta_{0}-\eta_{\min}\right)\left(1+\cos\left(\frac{T_{\mathrm{cur}}}{T_{\max}}\pi \right)\right),$$

where $\eta_{0}=1.25\times 10^{-4}$ is the initial learning rate, $\eta_{\min}=1\times 10^{-5}$ is the minimum learning rate, $T_{\max}$ is the total number of epochs, and $T_{\mathrm{cur}}$ is the current epoch.

The differences in training configuration stem from variations in dataset properties. For example, the *Drosophila* dataset contains high-resolution images (up to 2160×3500 pixels) with small targets, necessitating larger input size, smaller batch size, and longer training. In contrast, *C. elegans* (1024×1024) and zebrafish (855×750) datasets contain larger targets, allowing for larger batch sizes and shorter training schedules. Moreover, different keypoint configurations and grouping strategies across datasets require task-specific adaptations.

Testing: During inference, the short side of each image is resized to 512 for the *C. elegans* and zebrafish datasets, and 640 for the *Drosophila* dataset, while preserving aspect ratio. We perform single-scale testing with horizontal flipping.

Evaluation Metric: To evaluate keypoint detection performance, we use Average Precision (AP) and Average Recall (AR) based on Object Keypoint Similarity (OKS), as defined in Equation (9).

(9) $$\mathrm{OKS}=\frac{\sum_{i}\exp\left(-\frac{d_{i}^{2}}{2s^{2}k_{i}^{2}}\right)\cdot \sigma \left(v_{i}>0\right)}{\sum_{i}\sigma \left(v_{i}>0\right)}$$

Here, $d_{i}$ is the Euclidean distance between the *i*-th predicted and ground truth keypoint, $k_{i}$ is a keypoint-specific constant, *s* denotes object scale, $v_{i}$ is the visibility tag, and σ(·) is an indicator function returning 1 if the condition is true and 0 otherwise.

We report multiple AP metrics, including the mean Average Precision (AP) averaged over all OKS thresholds, ${\mathrm{AP}}^{50}$ at an OKS threshold of 0.5, and ${\mathrm{AP}}^{75}$ at an OKS threshold of 0.75. These metrics jointly evaluate the model’s keypoint localization accuracy under different precision requirements, where higher values indicate more precise predictions. In addition, Average Recall (AR) reflects the model’s ability to comprehensively and accurately detect all keypoints.

In addition to accuracy metrics, we evaluate the computational efficiency of each model using two indicators: Frames Per Second (FPS) and GFLOPs (Giga Floating Point Operations). FPS measures the real-time inference speed under a fixed resolution and batch size on an NVIDIA RTX 3090 GPU, directly reflecting the runtime performance. GFLOPs quantifies the theoretical computational complexity required for a single forward pass, providing a hardware-agnostic measure of model efficiency. These metrics enable a comprehensive assessment of the trade-off between accuracy and efficiency across species and model architectures.

## 5. Experiments

### 5.1. C. elegans Dataset

The *C. elegans* dataset was acquired from onboard cultivation experiments conducted aboard the China Space Station. To facilitate locomotor behavior analysis under microgravity conditions, five anatomically meaningful keypoints were defined to represent the worm’s pose, as illustrated in Figure 2a. The dataset consists of 6996 annotated images, including 5622 images with 12,349 individual worms in the training set and 1374 images with 3029 individuals in the test set. Each image has a resolution of 1024×1024 pixels. As the first dedicated pose estimation dataset for *C. elegans*, it provides a valuable foundation for quantitative behavioral studies, enabling the extraction of time-series motion trajectories and the computation of behavioral metrics such as head and tail swing frequencies.

Despite its utility, the dataset presents several challenges for pose estimation: (1) *C. elegans* exhibits a highly flexible and non-rigid body without a skeletal structure, resulting in diverse and unstable postures that are difficult to capture with conventional pose models; (2) multiple individuals frequently appear in overlapping or occluded scenes, which complicates the accurate association of keypoints; and (3) the worms’ visual similarity and lack of distinctive texture further hinder the precise localization of keypoints.

We compare our proposed method against several state-of-the-art pose estimation approaches, including AE [31], DEKR [36], CID [39], and AdaptivePose [38]. As shown in Table 1, our method outperforms all compared methods in both AP and AR metrics, achieving 0.728 and 0.793, respectively, which is attributed to our Multi-scale Feature Sampling (MFS) and Structure-guided Learning (SGL) modules.

**Figure 5:**
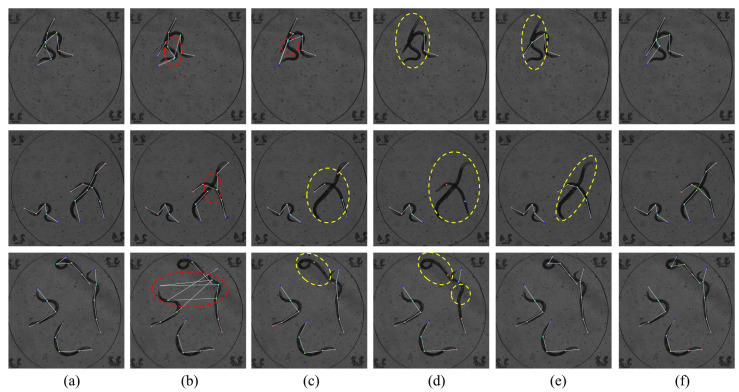
Visualization examples on the *C. elegans* dataset. Each row shows one sample image; columns represent different methods. From left to right: (**a**) Ground truth, (**b**) AE, (**c**) DEKR, (**d**) CID, (**e**) AdaptivePose, and (**f**) Ours. Red dashed boxes denote false positives, and yellow dashed boxes denote missed detections.

Figure 5 presents visualization results on the *C. elegans* dataset. Dots indicate keypoints, and white solid lines represent connections between them. We select three representative samples with two, three, and four individuals, respectively, each showing varying postures and levels of occlusion. Figure 5b shows the AE results, where red dashed boxes indicate misdetections due to difficulty distinguishing individuals in occluded scenes. Figure 5c shows the DEKR results, with false positives in the top sample and missed detections in the bottom two. Figure 5d shows the CID results, which exhibit significant missed detections in complex poses and occluded areas. Figure 5e shows AdaptivePose results with partial missed detections. Figure 5f displays the results of our method, which achieves the most accurate predictions even under heavy occlusion and overlaps, with predictions closely matching ground truth annotations.

### 5.2. Zebrafish Dataset

The zebrafish dataset was collected from ground-based experiments using the closed aquatic ecological experimental device designed for the China Space Station. It comprises a total of 560 annotated images, with 448 images (1790 instances) assigned to the training set and 112 images (447 instances) to the test set. Each image has a resolution of 855 × 750 pixels. To account for the zebrafish’s diverse postural features under varying observation angles, we define 10 keypoints to represent its skeletal structure (as illustrated in Figure 2b). This dataset presents considerable challenges for pose estimation: (1) zebrafish exhibit highly variable and flexible postures, making accurate keypoint prediction difficult; and (2) the image quality is often suboptimal, with frequent occurrences of blurriness, glare, and low illumination, which further hinder precise localization of keypoints.

Benefiting from the integration of the proposed Multi-scale Feature Sampling (MFS) module and Structure-guided Learning (SGL) module, our method demonstrates superior performance compared to existing baselines. As shown in Table 2, it achieves the highest scores in both accuracy and recall, with an Average Precision (AP) of 0.621 and an Average Recall (AR) of 0.664, surpassing state-of-the-art pose estimation models. These results highlight the effectiveness of our framework in addressing the challenging pose estimation task on the zebrafish dataset.

**Figure 6:**
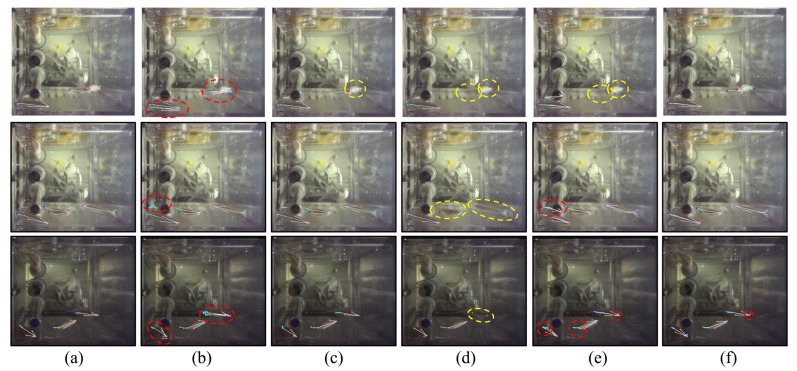
Visualization results of zebrafish pose estimation. Each row shows a sample image under different lighting conditions. From left to right: (**a**) ground truth, (**b**) AE, (**c**) DEKR, (**d**) CID, (**e**) AdaptivePose, and (**f**) our method. Red dashed lines indicate incorrect detections, and yellow dashed lines indicate missed detections.

Figure 6 presents qualitative visualization of pose estimation under three representative lighting conditions. Compared with other methods, our approach produces more robust predictions even in low-quality settings such as blurred images, strong reflections, and complex backgrounds. Specifically, AE (Figure 6b) produces numerous incorrect and redundant keypoint detections, as marked by red dashed lines. DEKR (Figure 6c) performs slightly better but still misses some keypoints (yellow dashed line). CID (Figure 6d) exhibits multiple missed detections, which is consistent with its lower AR value in Table 2. AdaptivePose (Figure 6e) shows variable performance with both missed and erroneous keypoints. In contrast, our method (Figure 6f) provides the most accurate and complete results, with only a minor misprediction at the head region in one example, further affirming the robustness of the proposed framework.

### 5.3. Drosophila Dataset

**Figure 7:**
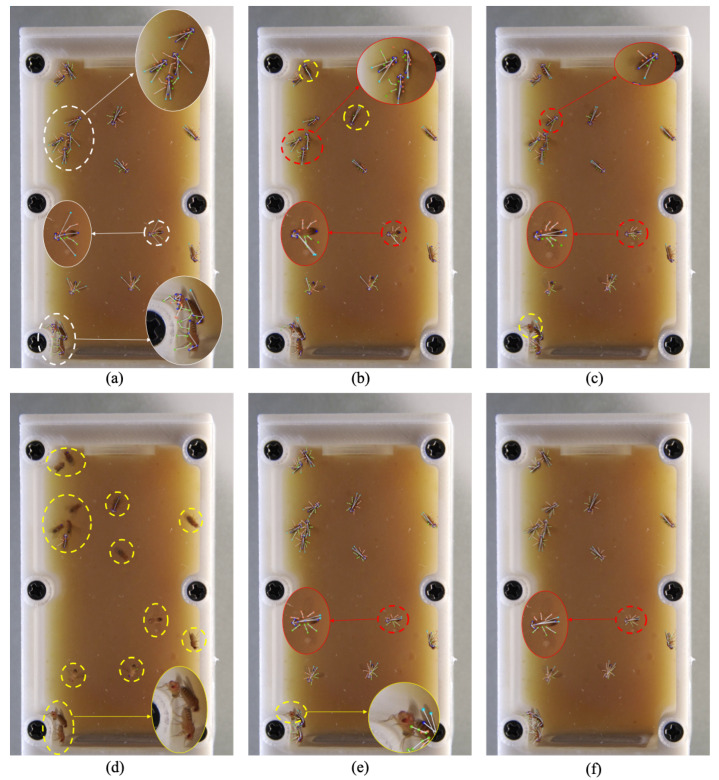
Visualization results on the *Drosophila* dataset. (**a**) shows ground-truth annotations; (**b**–**f**) show predictions from AE, DEKR, CID, AdaptivePose, and our method, respectively. Red dashed lines indicate false positives, and yellow dashed lines indicate missed detections. Zoomed-in regions highlight areas with key differences.

The *Drosophila* dataset was collected from ground-based experiments using the fruit fly culture system designed for the China Space Station. A total of 410 images were annotated, including 328 training images (containing 3310 individuals) and 82 testing images (containing 1076 individuals). The image resolution ranges from 1200 × 1970 to 2160 × 3500 pixels. Considering the demands of behavior analysis on localization accuracy and image clarity, we define 26 keypoints to represent the pose of each fly (as shown in Figure 2c). As illustrated in Figure 7, this dataset presents several challenges: (1) The experimental scene contains a large number of visually similar individuals with frequent occlusions, making pose estimation highly difficult. (2) The small body size of fruit flies and the use of 4K imaging for high-resolution recording pose high demands on fine-grained feature extraction.

Beyond *C. elegans* and zebrafish, our method is also successfully applied to estimate the poses of *Drosophila* individuals in high-density 4K imagery. As shown in Table 3, our approach significantly outperforms existing methods, achieving an AP of 0.671 and an AR of 0.732. Compared to heatmap-based methods, such as DEKR and CID, our regression-based method improves accuracy by 8.8% and 10.9%, respectively. This is mainly due to the reduced loss of small-part features (e.g., legs) caused by downsampling in heatmap methods. Furthermore, our method achieves the highest AR, indicating a lower miss rate across individuals.

As shown in Figure 7, we present representative close-up areas with high fly density and complex postures, including leg contact and wing spread. Existing multi-animal pose estimation methods are typically evaluated on at most two flies per frame, whereas our dataset contains up to 10 individuals per frame, covering group behaviors and various angles—posing greater challenges. As seen in the visualizations, methods like CID suffer from significant miss detection (Figure 7d), failing to generate valid poses. Some methods poorly localize small parts like legs and wings (Figure 7b,c). AdaptivePose (Figure 7e) performs relatively well but still exhibits mis- and missed detections. Our method (Figure 7f) performs stably under crowded conditions with fewer errors, although wing localization under spread remains improvable.

## 6. Ablation Study

In this section, we conduct comprehensive ablation experiments on three representative space animal datasets—*C. elegans*, zebrafish, and *Drosophila*—to evaluate the effectiveness of the proposed modules. All experiments are performed under the same data settings and experimental environment.

### 6.1. Ablation Study on the Multi-Scale Feature Sampling Module (MFS)

This subsection investigates the design choices within the Multi-scale Feature Sampling Module (MFS). The baseline model is based on the AdaptivePose architecture, supervised by a combination of penalty FocalLoss (for center and keypoint heatmaps) and L1Loss (for keypoint offset). The baseline utilizes only the downsampled feature map $F_{1/4}$ as input for downstream modules. In contrast, our MFS module extracts multi-scale features ($F_{ms}$) from HRNet and incorporates three components: channel transformation (Ch-trans Block), feature resampling (Sampling Block), and adaptive feature fusion (Adaptive Fusion Block).

Two Ch-trans strategies are tested: CT1 applies a sequential conv2d–ReLU–conv2d transformation, while CT2 enhances spatial adaptability by introducing deformable convolutions via conv2d–ReLU–$conv_{bn}$–ReLU–DCN. The Sampling Block mitigates semantic distortion caused by scale variation, and the fusion strategy is implemented in two forms: direct concatenation and adaptive addition ($A_{add}$). We conduct experiments combining different feature sets, including $F_{1/4},\;F_{1/8},\;F_{1/16},$ and $F_{1/32}$. The results are presented in Table 4.

As shown in Table 4, the choice of channel transformation, fusion strategy, and feature scale all significantly impact performance. Notably, $A_{add}$ outperforms direct concatenation, and the CT2 strategy with deformable convolutions is superior to the simple convolutional stack in CT1. Furthermore, combining features from multiple resolutions yields better results than relying solely on $F_{1/4}$, demonstrating the value of high-level features in enhancing multi-scale representation. However, over-fusion with excessively downsampled features (e.g., $F_{1/32}$) slightly degrades accuracy, likely due to the loss of spatial precision. Ultimately, the combination of $CT2+A_{add}+\left[F_{1/4},F_{1/8}\right]$ is selected as the final MFS design, striking a balance between accuracy and computational efficiency.

### 6.2. Ablation Study on Structure-Guided Learning Module (SGL)

This section evaluates the effectiveness of the proposed Structure-guided Learning (SGL) module. The module introduces a multi-head self-attention mechanism to capture the structural priors of animal parts, which enhances the pose estimation performance under complex conditions, such as occlusion and entanglement. The ablation focuses on three components: whether to include part type embeddings (Type Embed), the dimensionality of the feedforward network (Dim_Feedforward), and the number of Transformer encoder layers (Num_Layers). The experimental results on the *C. elegans* dataset are shown in Table 5.

The results indicate that incorporating part-type embeddings significantly improves performance, likely due to better modeling of region-specific priors. Increasing the feedforward dimensionality to 1024 results in performance degradation, which suggests that higher dimensions may lead to overfitting or unstable optimization. Additionally, using more Transformer layers does not guarantee improved results—adding a second layer yields slight gains in AP and AR but remains inferior to the simpler configuration with one layer. Therefore, the optimal configuration adopted in subsequent experiments consists of enabling type embeddings, using a feedforward dimension of 512, and applying a single Transformer encoder layer.

**Figure 8:**
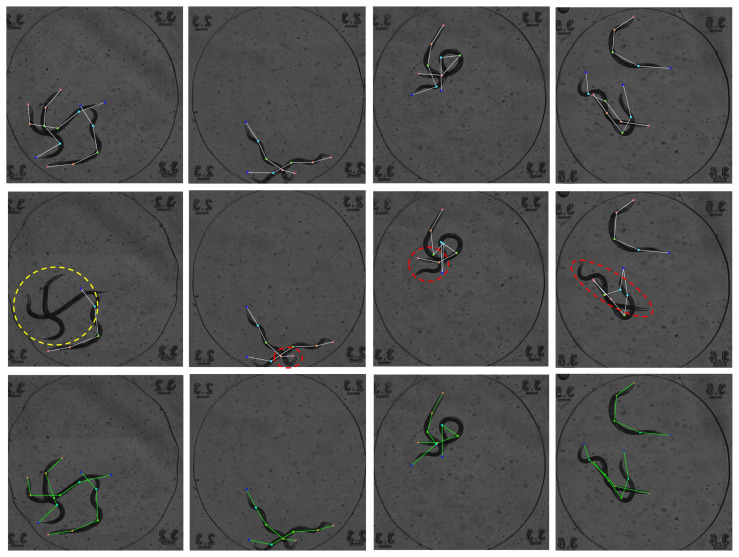
Comparison of pose estimation results between the baseline and our model with the Structure-guided Learning Module. From top to bottom: ground truth labels, baseline prediction, and prediction from our method. Red dashed boxes indicate incorrect predictions; yellow dashed boxes indicate missed detections.

To further demonstrate the practical benefits of the SGL module, Figure 8 presents a qualitative comparison between the baseline model and our improved version. From top to bottom, the figure shows the ground truth annotations, results from the baseline method, and results from our model incorporating the SGL module. The red dashed boxes highlight instances of incorrect keypoint estimation, while the yellow dashed boxes indicate missed detections. The baseline model struggles in complex scenarios with dense occlusion or body overlap, often leading to partial or incorrect pose predictions. In contrast, the SGL-enhanced model is able to recover more complete and structurally accurate poses. This validates that our approach enables the network to leverage structural dependencies between body parts, which is especially beneficial for scenes with heavy occlusion or ambiguous posture, thereby improving overall pose estimation robustness.

### 6.3. Ablation Experiments on Different Modules Across Three Datasets

To evaluate the effectiveness of each proposed module, we conduct ablation experiments on three datasets: *C. elegans*, zebrafish, and *Drosophila*. As shown in Table 6, we assess the performance of the baseline, the Multi-scale Feature Sampling (MFS) module, the Structure-guided Learning (SGL) module, and the combination of both (+MFS&SGL).

From Table 6, it can be observed that incorporating either the Multi-Scale Feature Sampling (MFS) or the Structure-Guided Learning (SGL) module consistently improves performance across all three datasets. The combination of both modules achieves the best overall results in terms of AP and AR, highlighting their complementary strengths. On the *C. elegans* and *Drosophila* datasets, the standalone SGL module attains slightly higher ${\mathrm{AP}}^{75}$ than the combined configuration, suggesting that the high-level semantic aggregation introduced by MFS may marginally influence fine-grained keypoint localization. Nevertheless, MFS significantly enhances global contextual perception and improves robustness under varying scales and occlusions, leading to the highest overall AP when integrated with SGL.Specifically, compared with the baseline, the combined model achieves AP improvements of 4.3%, 4.4%, and 5.1% on the *C. elegans*, zebrafish, and *Drosophila* datasets, respectively, demonstrating the effectiveness of jointly leveraging structural and multi-scale information.

In addition, from the perspective of model complexity, introducing the Multi-Scale Feature Sampling (MFS) module results in only a marginal increase in computational cost (+1.7 GFLOPs on the *C. elegans* dataset, +1.7 GFLOPs on the zebrafish dataset, and +1.2 GFLOPs on the *Drosophila* dataset), while achieving consistent performance improvements across all datasets (AP gains of +3.2%, +3.2%, and +2.2%, respectively). These results indicate that the MFS module substantially enhances feature adaptability to multi-scale structures without compromising efficiency.

### 6.4. Effect of Removing Keypoint Heatmap Branch During Inference

To evaluate the impact of removing the Keypoint Heatmap Branch during inference, we conducted an ablation experiment on the *C. elegans* dataset. This branch is only used during training to guide the learning of spatial priors for keypoint localization and is removed at test time to reduce computational cost. As shown in Table 7, removing this branch during inference has a negligible effect on the final accuracy, confirming its auxiliary role.

### 6.5. Efficiency Comparison Across Different Frameworks

To evaluate the computational efficiency and accuracy of different frameworks on multi-species space animal data, we compared representative top–down, bottom–up, and one-stage methods on the *C. elegans*, zebrafish, and *Drosophila* datasets. The results are summarized in Table 8.

As shown in Table 8, conventional top–down methods such as HRNet [26] perform pose estimation within a two-stage pipeline, where each instance is first localized by a detector and then cropped for keypoint prediction. Although this strategy achieves relatively high accuracy, it incurs substantial computational overhead and leads to slow inference, especially in dense multi-animal scenes. Bottom–up methods (e.g., AE) enable full-image parallel processing but still require complex post-processing for keypoint grouping, which limits their real-time applicability. In contrast, one-stage approaches unify instance localization and keypoint regression into a single feed-forward pass, offering a significantly better trade-off between accuracy and efficiency. The proposed method achieves the highest AP on all three datasets (72.8%, 62.1%, and 67.1%) while maintaining real-time inference speeds of 12.66, 11.24, and 6.02 FPS, respectively. These results demonstrate the superior accuracy–efficiency balance and strong cross-species adaptability of the proposed single-stage framework.

## 7. Discussion

The proposed two-hop regression-based single-stage pose estimation framework demonstrates outstanding performance on the challenging SpaceAnimal dataset, particularly under complex scenarios characterized by high individual density, severe occlusions, and substantial interspecies morphological differences. Compared to conventional two-stage methods, our approach achieves a better balance between accuracy and efficiency. As shown in Table 8, our method consistently achieves the highest average precision (AP) and average recall (AR) across three model organisms—*C. elegans*, zebrafish, and *Drosophila*—while maintaining high inference speed, effectively addressing both accuracy and efficiency requirements.

Comparative experiments further reveal that existing methods exhibit species-specific advantages: CID performs best on *C. elegans* (Table 1), DEKR excels in zebrafish pose estimation (Table 2), and Adaptive Pose is more suitable for *Drosophila*’s complex body structures (Table 3). In contrast, our proposed method is the only one that achieves the best keypoint detection accuracy across all three species, indicating strong cross-species generalization and structure-aware modeling capabilities.

In terms of efficiency, traditional top–down methods rely on a pre-processing object detection module, while bottom–up methods involve complex post-processing procedures, such as keypoint grouping and clustering, which become bottlenecks in multi-instance scenarios. Our single-stage framework eliminates intermediate processing, resulting in a significant speed-up: on *C. elegans* (1–4 instances), it is approximately 3.4× faster than two-stage methods (12.66 FPS vs. 3.70 FPS); on zebrafish (4 instances), about 4.2× faster (11.24 FPS vs. 2.67 FPS); and on *Drosophila* (9–15 instances), about 5.9× faster (6.02 FPS vs. 1.02 FPS). This performance suggests the potential for real-time onboard deployment and lays a foundation for subsequent behavior analysis tasks.

Nonetheless, the current framework has several limitations: (1) Species Dependency: The current keypoint grouping strategy is manually defined based on species-specific structural priors. While effective under strong supervision, it lacks generalizability to new species or individuals with significant morphological variations. Future work may consider integrating language models or body-part-based semantic descriptions to construct more flexible and generalizable grouping mechanisms. (2) Lack of Temporal Modeling: Our method currently operates on single-frame images, making it unsuitable for behavior analysis tasks that rely on temporal dynamics—such as head–tail oscillation frequency in *C. elegans*, disorientation behaviors in zebrafish (e.g., circling, inverted swimming), or motor impairment in *Drosophila* (e.g., misaligned pose-motion direction or uncontrolled spinning). Incorporating temporal modeling modules, such as temporal convolution or transformer-based architectures, would enhance its robustness for dynamic behavior recognition.

In summary, the proposed method achieves high-precision and high-efficiency keypoint detection across multiple species in the SpaceAnimal dataset. It provides a foundational tool for contact-free, automated behavior analysis in space life science experiments. Future work may optimize the model for two major application scenarios: (1) scientific analysis, where accuracy and representational capacity are prioritized to support high-quality behavioral phenotyping; and (2) real-time onboard monitoring, where lightweight design, parallel computing, and compatibility with in-orbit computational resources are crucial, facilitating a transition from “observable” to “quantifiable” and “explainable” space animal behavior analysis.

## 8. Conclusions

In this paper, we propose a single-stage multi-animal pose estimation method tailored for diverse spatial animals under microgravity conditions. Extensive experiments conducted on three distinct datasets—*C. elegans*, zebrafish, and *Drosophila*—demonstrate that our method achieves competitive performance compared to state-of-the-art approaches. The introduced Multi-scale Feature Sampling (MFS) module enables the network to effectively capture high-resolution local details alongside semantically rich global features. By adaptively sampling and fusing multi-scale features, the model shows strong generalization ability across species and varying body sizes. Meanwhile, the Structure-guided Learning (SGL) module reinforces the learning of structural priors by modeling spatial dependencies among body parts, which enhances robustness against pose variability and occlusion. Quantitative results confirm the superior performance of our method across all benchmarks. In future work, we plan to integrate temporal modeling and inter-frame association mechanisms to extract fine-grained motion cues and enable pose tracking. Furthermore, this framework can support comparative analysis between spaceflight and ground-based experiments, offering valuable insights into animal behavior under microgravity. To better meet the personalized requirements of biological experiments, future extensions may also explore large model-based pose estimation and language-guided keypoint selection strategies.

## Figures and Tables

**Table 1:** Evaluation results on the *C. elegans* dataset. Our method achieves the best performance in both AP and AR compared to previous methods.

Method	Input Size	Backbone	AP	${\mathrm{AP}}^{50}$	${\mathrm{AP}}^{75}$	AR
AE	512 × 512	HRNet-W32	0.657	0.951	0.723	0.733
DEKR	512 × 512	HRNet-W32	0.633	0.889	0.717	0.691
CID	512 × 512	HRNet-W32	0.703	0.947	0.796	0.763
AdaptivePose	512 × 512	HRNet-W32	0.685	0.936	0.768	0.757
Ours	512 × 512	HRNet-W32	0.728	0.970	0.807	0.793

**Table 2:** Evaluation results on the zebrafish dataset. Our method achieves the best performance in terms of both accuracy and recall. The best results are highlighted in bold.

Method	Input Size	Backbone	AP	AP^50^	AP^75^	AR
AE	512 × 512	HRNet-W32	0.562	0.770	0.617	0.438
DEKR	512 × 512	HRNet-W32	0.591	0.780	0.655	0.421
CID	512 × 512	HRNet-W32	0.575	0.765	0.665	0.637
AdaptivePose	512 × 512	HRNet-W32	0.577	0.788	0.632	0.624
**Ours**	512 × 512	HRNet-W32	**0.621**	**0.804**	**0.679**	**0.664**

**Table 3:** Evaluation results on the *Drosophila* dataset. Our method achieves the best performance compared to state-of-the-art methods. The best scores are highlighted in bold.

Method	Input Size	Backbone	AP	${\mathrm{AP}}^{50}$	${\mathrm{AP}}^{75}$	AR
AE	640 × 640	HRNet-W32	0.469	0.776	0.529	0.536
DEKR	640 × 640	HRNet-W32	0.583	0.906	0.667	0.633
CID	640 × 640	HRNet-W32	0.562	0.853	0.655	0.623
Adaptive Pose	640 × 640	HRNet-W32	0.620	0.909	0.677	0.688
Ours	640 × 640	HRNet-W32	**0.671**	**0.951**	**0.740**	**0.732**

**Table 4:** Ablation results of the Multi-scale Feature Sampling Module (MFS) on the *C. elegans* dataset. Best results are highlighted in bold.

Ch_trans	Fusion	Features	AP	${\mathrm{AP}}^{50}$	${\mathrm{AP}}^{75}$	AR
CT1	con_cat	$F_{1/4},F_{1/8},F_{1/16},F_{1/32}$	0.694	0.948	0.772	0.756
CT1	A_add	$F_{1/4},F_{1/8},F_{1/16},F_{1/32}$	0.705	0.953	0.789	0.770
CT2	A_add	$F_{1/4},F_{1/8},F_{1/16},F_{1/32}$	0.708	0.949	0.786	0.774
CT2	A_add	$F_{1/4},F_{1/8},F_{1/16}$	**0.717**	0.965	**0.811**	**0.783**
CT2	A_add	$F_{1/4},F_{1/8}$	**0.717**	**0.967**	0.792	**0.783**
CT2	A_add	$F_{1/4}$	0.691	0.945	0.769	0.761
CT2	A_add	$F_{1/4},F_{1/16}$	0.713	0.964	0.808	0.780
CT2	A_add	$F_{1/4},F_{1/32}$	0.711	0.975	0.795	0.785

**Table 5:** Ablation results of the Structure-guided Learning (SGL) module on the *C. elegans* dataset. Best results are highlighted in bold.

Type Embed	Dim_Feedforward	Num_Layers	AP	${\mathrm{AP}}^{50}$	${\mathrm{AP}}^{75}$	AR
Disabled	512	1	0.706	0.955	0.792	0.777
Enabled	512	1	**0.718**	**0.969**	**0.817**	**0.788**
Enabled	1024	1	0.711	0.945	0.678	0.755
Enabled	512	2	0.714	0.964	0.806	0.782

**Table 6:** Ablation study of the proposed method on the three datasets. The best result is highlighted in bold.

Dataset	Method	Input	GFLOPs	Params/M	AP	${\mathrm{AP}}^{50}$	${\mathrm{AP}}^{75}$	AR
*C. elegans*	Baseline	512	52.24	29.4	0.685	0.936	0.768	0.757
	+MFS	512	53.94	31.43	0.717	0.967	0.792	0.783
	+SGL	512	57.65	29.48	0.718	0.969	**0.817**	0.788
	+MFS&SGL	512	59.35	31.50	**0.728**	**0.970**	0.807	**0.793**
Zebrafish	Baseline	512	52.25	29.4	0.577	0.788	0.632	0.624
	+MFS	512	53.95	31.43	0.609	0.796	0.648	0.655
	+SGL	512	57.66	29.48	0.607	0.792	0.659	0.650
	+MFS&SGL	512	59.36	31.50	**0.621**	**0.804**	**0.679**	**0.664**
*Drosophila*	Baseline	640	58.44	29.93	0.620	0.909	0.677	0.688
	+MFS	640	59.64	31.94	0.642	0.921	0.719	0.704
	+SGL	640	68.18	30.01	0.666	0.946	**0.757**	0.728
	+MFS&SGL	640	69.88	32.03	**0.671**	**0.951**	0.740	**0.732**

**Table 7:** Ablation on removing the Keypoint Heatmap Branch during inference on the *C. elegans* dataset.

Method	AP (%)	AR (%)
Ours (with heatmap branch during inference)	72.91	79.42
Ours (heatmap branch removed during inference)	72.83	79.30

**Table 8:** Inference efficiency comparison of different methods on three space animal datasets.

Dataset	Method Type	Method	Input Size	AP	FPS
*C. elegans*	Top–Down	HRNet	256 × 192	0.701	2.98
	Bottom–Up	AE	512 × 512	0.657	3.70
	One-Stage	Ours	512 × 512	0.728	12.66
Zebrafish	Top-Down	HRNet	256 × 192	0.537	2.27
	Bottom-Up	AE	512 × 512	0.562	2.76
	One-Stage	Ours	512 × 512	0.621	11.24
*Drosophila*	Top-Down	HRNet	256 × 192	0.608	0.72
	Bottom-Up	AE	640 × 640	0.399	1.02
	One-Stage	Ours	640 × 640	0.671	6.02

## Data Availability

The SpaceAnimals datasets presented in this study can be accessed at the following link: https://doi.org/10.1038/s41597-025-05111-8.
